# Milk sharing: from private practice to public pursuit

**DOI:** 10.1186/1746-4358-6-8

**Published:** 2011-06-25

**Authors:** James E Akre, Karleen D Gribble, Maureen Minchin

**Affiliations:** 1 Geneva, Switzerland; 2 School of Nursing and Midwifery, University of Western Sydney, Locked Bag 1797, Penrith, New South Wales, 2751. Australia; 3 Geelong, Victoria, Australia

## Abstract

After only six months, a commerce-free internet-based milk-sharing model is operating in nearly 50 countries, connecting mothers who are able to donate breast milk with the caregivers of babies who need breast milk. Some public health authorities have condemned this initiative out of hand. Although women have always shared their milk, in many settings infant formula has become the "obvious" alternative to a mother's own milk. Yet an internationally endorsed recommendation supports mother-to-mother milk sharing as the best option in place of a birth mother's milk. Why then this rejection? Several possibilities come to mind: 1) ignorance and prejudice surrounding shared breast milk; 2) a perceived challenge to the medical establishment of a system where mothers exercise independent control; and 3) concern that mother-to-mother milk sharing threatens donor milk banks. We are not saying that milk sharing is risk-free or that the internet is an ideal platform for promoting it. Rather, we are encouraging health authorities to examine this initiative closely, determine what is happening, and provide resources to make mother-to-mother milk sharing as safe as possible. Health authorities readily concede that life is fraught with risk; accordingly, they promote risk-reduction and harm-minimisation strategies. Why should it be any different for babies lacking their own mothers' milk? The more that is known about the risks of substituting for breast milk, the more reasonable parental choice to use donor milk becomes. We believe that the level of intrinsic risk is manageable through informed sharing. If undertaken, managed and evaluated appropriately, this made-by-mothers model shows considerable potential for expanding the world's supply of human milk and improving the health of children.

## Background

We are watching with fascination, admiration and anxiety as mother-to-mother human-milk sharing goes global via the internet [1,2]. Fascination, because this ageless and largely private practice has suddenly burst into the public arena as a topic of unprecedented discussion, both amongst individuals, and in the popular media. Admiration, because it takes self-help and female solidarity to new heights, illustrating how women are innovating to tackle breast milk scarcity and deny infant formula its default-substitute status. Anxiety, because some public health authorities, notably in Canada, France and the USA, are condemning it out of hand [3-5].

This contemporary variation on a practice that is as old as our species [2] warrants a closer look than that implied by a "just say no" summary dismissal. After only six months under the exclusive influence of mothers interacting with other mothers, internet-based milk sharing - as exemplified by two sites, Eats On Feets and Human Milk 4 Human Babies [2] - is already operating in nearly 50 countries. Both use Facebook to connect mothers under a commerce-free model in which milk is not bought and sold but is freely given. Well-informed and highly motivated women have begun extending control over the availability and use of human milk, and it is improbable they will be deterred by unsupportive or critical public health authorities. Indeed, they await no one's permission - the milk donors in ensuring the availability of milk for children other than their own, and the milk recipients in meeting the nutritional needs of children who would otherwise be fed artificially (i.e. with infant formula).

## Milk sharing on the internet

In many settings breast milk and breastfeeding have been undervalued, and the nutritional merits and safety of infant formula exaggerated (for an in depth discussion of this issue see Hausman [6]). The result: infant formula is considered the "obvious" alternative to a mother's own milk. However, the international infant feeding recommendation in place for the past 25 years describes a different nutritional hierarchy for babies who are not fed at the breast but for whom breast milk remains the food of choice: expressed breast milk provided by their own mothers, followed by breast milk from a wet-nurse, or from a breast-milk bank [7]. Additionally, in 2002 the World Health Organization declared that: "For those few health situations where infants cannot, or should not, be breastfed, the choice of the best alternative - expressed breast milk from an infant's own mother, breast milk from a healthy wet-nurse or a human-milk bank, or a breast-milk substitute fed with a cup ... depends on individual circumstances" [8]. Caregivers of infants may seek peer-to-peer donor milk in order to avoid the risks inherent to formula feeding.

So, if an internationally endorsed recommendation already exists for mother-to-mother milk sharing as the best alternative to a birth mother's own milk, why the rejection of this internet-based model? Several possibilities come to mind.

Firstly, there is a long history of suspicion surrounding women's milk. For centuries people worried about the impact on breastfed children of the milk provider's physical traits, morals, lifestyle, diet and health [9]. Suspicion persists today with a focus on possible transmission of infection by breast milk [10], which is the principal concern raised about the internet-based groups. Whilst concerns about the safety of sharing milk would exist regardless of the mode of sharing, the fact that the internet is being used to facilitate such milk sharing seems to enhance these fears.

Secondly, this internet-based model could be perceived as a challenge to the medical establishment: a system that operates independently of its influence, that cannot be regulated, and where mothers alone exercise control. This would not be the first time that breastfeeding mothers have exasperated health authorities. The originators of the early organised mother-to-mother breastfeeding support networks, for example La Leche League and the Australian Breastfeeding Association, were also dismissed for advocating such "radical" practices [11] - all widely followed today consistent with a strong evidence base - as rooming-in, breastfeeding on demand, and no test-weighing, which put mothers, not health professionals, in charge. It may be that as time passes, and as an evidence base is built for peer-to-peer milk sharing, that concerns about mother's control of milk sharing will similarly recede. This is perhaps more likely if authorities can collaborate with mothers to provide information that assists quality milk-sharing regimes on the internet. However, this suspicion of mothers and their milk is something that has been previously identified [10].

Finally, some observers appear to be concerned that mother-to-mother milk sharing threatens supply for the relatively few donor human-milk banks. However, this objection would not hold up if, in fact, mother-to-mother milk sharing involves different groups of mothers and babies. Babies receiving banked donor milk appear to be almost exclusively the sick and the hospitalised [12,13], whereas those receiving milk mother-to-mother are children who do not usually qualify for banked donor milk. Similarly, given milk banks' exclusion criteria - for example: previous residence in the United Kingdom (due to possible infection with Creutzfeldt-Jakob disease), regular consumption of caffeinated beverages, a baby older than six months, and only a small amount of available milk - many women willing to donate their milk to banks are unable to. Thus, mother-to-mother milk sharing should be viewed as complementary to donor-milk banking and not as its competitor. Moreover, the expanding network of mother-to-mother milk sharing might well spur human-milk banking by increasing awareness of the significance and availability of breast milk, persuading more qualifying mothers to donate, and thereby increasing both the number of banks and the available milk volume. However, some involved in human-milk banking are concerned that any case of disease transmission via peer-to-peer human-milk sharing could have negative consequences for human milk banks, many of which have struggled against uninformed decision-makers in order to establish their operations. This concern is a real one. Sadly, illness and death due to artificial feeding is rarely newsworthy, but any adverse outcomes arising from peer-to-peer human-milk sharing would be widely publicised. This concern underlines the need for guidance to make peer-to-peer milk sharing as safe as is possible. A response of denial and proscription is unlikely to help infants.

The obvious question that needs to be asked is this: Why do babies need breast milk from donors? In some situations, donor breast milk is the only option, for example where the mother has had a double mastectomy or has died. In others, however, low maternal milk supply has a social rather than a physiological origin, including when mothers are required to return to workplaces that do not allow either breastfeeding or milk expression, or where unrealistic expectations about the reasonable frequency of breastfeeding results in insufficient milk production. Certainly, the availability of donor breast milk does nothing to dilute the imperative of eliminating barriers to mothers breastfeeding their own babies. Providing breast milk is not the same as breastfeeding.

Nor are we saying that mother-to-mother milk sharing is risk-free, that the internet is an ideal platform for promoting it, or that milk donors and recipients alike can proceed without caution. The aim of helping mothers who are unable to supply enough milk to their children is no better served by naïve optimism than outright censure. Rather, acknowledging that, here as elsewhere, parents are responsible for making informed choices about what is best for their children, we encourage health authorities to determine what is happening, and then provide practical support to make mother-to-mother human-milk sharing as safe as possible.

Health authorities readily concede that life is fraught with risk; accordingly, they promote strategies to manage risk and minimise harm. For example, Health Canada educates about reducing the risk of powdered infant formula's intrinsic contamination with pathogenic bacteria [14]; and the US Food and Drug Administration alerts the public to infant formula products recalled for safety reasons [15]. In the light of the evidence [16], there is no such thing as risk-free infant-formula feeding. The more that is known about the risks of substituting for breast milk, the more reasonable parental choice to use donor milk becomes. Suitable approaches to minimising milk-sharing risk could include information on appropriate donor screening, reliable methods of exchange and feeding, and expediting voluntary sharing of medical records. Consideration of the process of peer-to-peer milk sharing and the methods by which it might be made as safe as possible should be the focus of future discussions of milk sharing.

## Conclusion

Mothers are leading in this initiative. The public health community has a choice: stay on the side-lines or move to engage, to assist those who are involved in milk sharing to make it as safe as possible. We appeal for engagement in the belief that milk sharing will happen regardless of denunciations; that its level of risk is manageable; and that there are greater intractable risks for babies who do not receive breast milk [16,17]. We believe that if undertaken, managed and evaluated appropriately, this made-by-mothers model shows considerable potential for expanding the world's supply of human milk and improving the health of children.

## Competing interests

The authors declare that they have no competing interests.

## Authors' contributions

JEA conceived the paper; JEA and KDG together drafted it. MM provided critical analysis and input. The authors have read and approved of the final manuscript.

## Authors' information

JEA is an author and commentator interested in the sociocultural impact on the universal biological norm for feeding children, and in pathways for returning breastfeeding and breast-milk feeding to the realm of the ho-hum ordinary everywhere.

KDG is a researcher on infant feeding and a breastfeeding advocate. She has an interest in wet nursing and cross nursing, and has previously published papers on adoptive breastfeeding and foster breastfeeding.

MM is an historian and widely published author, who has worked for decades to improve the level of health professional knowledge, attitudes, and practice in all areas of infant feeding. She is currently engaged in researching various neglected aspects of artificial feeding.
